# Recycling Waste Soot from Merchant Ships to Produce Anode Materials for Rechargeable Lithium-Ion Batteries

**DOI:** 10.1038/s41598-018-23945-8

**Published:** 2018-04-04

**Authors:** Won-Ju Lee, Han Vin Kim, Jae-Hyuk Choi, Gasidit Panomsuwan, Young-Chan Lee, Beom-Seok Rho, Jun Kang

**Affiliations:** 1 Division of Marine Engineering, Korea Maritime and Ocean University, Busan, 49112 Korea; 2 Division of Marine System Engineering, Korea Maritime and Ocean University, Busan, 49112 Korea; 3 Department of Materials Engineering, Faculty of Engineering, Kasetsart University, Bangkok, 10900 Thailand; 4 Division of Marine Information Technology, Korea Maritime and Ocean University, Busan, 49112 Korea; 5 Korea Institute of Maritime and Fisheries Technology, Beom-Seok Roh, Busan, 49111 Korea

## Abstract

In this study, the waste soot generated by ships was recycled to produce an active material for use in lithium-ion batteries (LIBs). Soot collected from a ship was graphitized by a heat treatment process and used as an anode active material. It was confirmed that the graphitized soot was converted into a highly crystalline graphite, and was found to form carbon nano-onions with an average diameter of 70 nm. The graphitized soot showed a high discharge capacity and an excellent cycle life, with a reversible capacity of 260 mAhg^−1^ even after 150 cycles at a rate of 1 C. This study demonstrates that the annealed soot with a unique graphitic multilayer structure has an electrochemical performance that renders it suitable as a candidate for the production of low-cost anode materials for use in LIBs.

## Introduction

Maritime transport handles over 80% of global trade^1^, and international shipping is a developing field in the world economy^2^. International trade has been growing since 2000^3–5^, and thus, the world fleet has gradually grown over the last decade^6^. This has led to significant increase in fuel consumption^7^ due to the increase in total engine power installed on ships^6^. In merchant ships, where diesel engines are largely used as prime movers^8–10^, most engines generate propulsion power from the combustion of low-quality heavy fuel oil (HFO)^11,12^, which emits polluting by-products^1,13,14^. The quantity of soot originating from ocean-going vessels is increasing because of the absolute correlation between fuel consumption due to international shipping and global maritime trade levels^15,16^.

On the other hand, revised MARPOL (the International Convention for the Prevention of Pollution from Ships) Annex V regulations forbid vessels from discharging a variety of garbage into the sea, including soot. However, it is difficult for shipping companies to dispose garbage such as soot, and there is no clear way to treat soot without landing ashore, which is expensive. Soot particles originating from marine diesel engines^17,18^ are emitted into the atmosphere via exhaust streams. According to international estimates, the annual quantity of exhaust particles emitted from shipping is between 0.9 and 1.7 million tons^19^. Some of them adhere to the heat transfer surfaces of the economizer, which reduces its efficiency and can have debilitating effects including increased cleaning cost, corrosion, and the risk of soot fires. It is difficult to correctly estimate the table.

Quantity of soot deposits that adhere to the economizer because it is immensely difficult to collect pollutants and emission data from ships in operation^20^. In case of an ocean-going 5,300 TEU (twenty-foot equivalent unit) container vessel, more than 1,000 litres/year of soot are normally collected when cleaning the economizer, and is taken ashore for disposal.

The main constituent of soot is carbon (>80 wt%)^21,22^, which is produced during high-temperature pyrolysis or combustion processes^22–24^ and is an inherent by-product of the operation of a compression ignition engine^25^. However, to date, no studies have tried to utilize soot, which contains nanostructures that show some degree of crystalline order^26^. In this work, an attempt to recycle waste soot from ships to provide an active material for use in lithium-ion batteries (LIBs) was made for the first time, which is a unique way of attempting to utilize waste to provide renewable energy. This is possible because graphite is often used as an active anode material in LIBs, and the soot generated by marine diesel engines is mostly composed of carbon and graphitic nanostructures.

Artificial graphite for use in LIBs is produced by first obtaining a carbon precursor, carbonizing it, and then graphitizing it to increase its crystallinity. In the case of graphite reformed from waste soot, such as that used in this study, precursor generation and carbonization processes are performed in a combustion engine, and only the graphitization process needs to be carried out. This can make this method much more cost effective than other methods of producing artificial graphite are.

In this study, soot samples collected from a marine diesel engine of an ocean-going vessel were analysed by high-resolution transmission electron microscopy (HRTEM), X-ray diffraction (XRD), Raman spectroscopy, and Brunauer-Emmett-Teller (BET) theory to investigate their structural characteristics. In order to improve the crystallinity of the soot to facilitate insertion of Li ions into the graphene layers, graphitization was conducted by annealing at 2700 °C. LIBs were manufactured using the annealed soot, and their electrochemical performances were evaluated to verify the possibility of using this material in anodes for LIBs.

## Experimental

### Material collection

**Figure 1:**
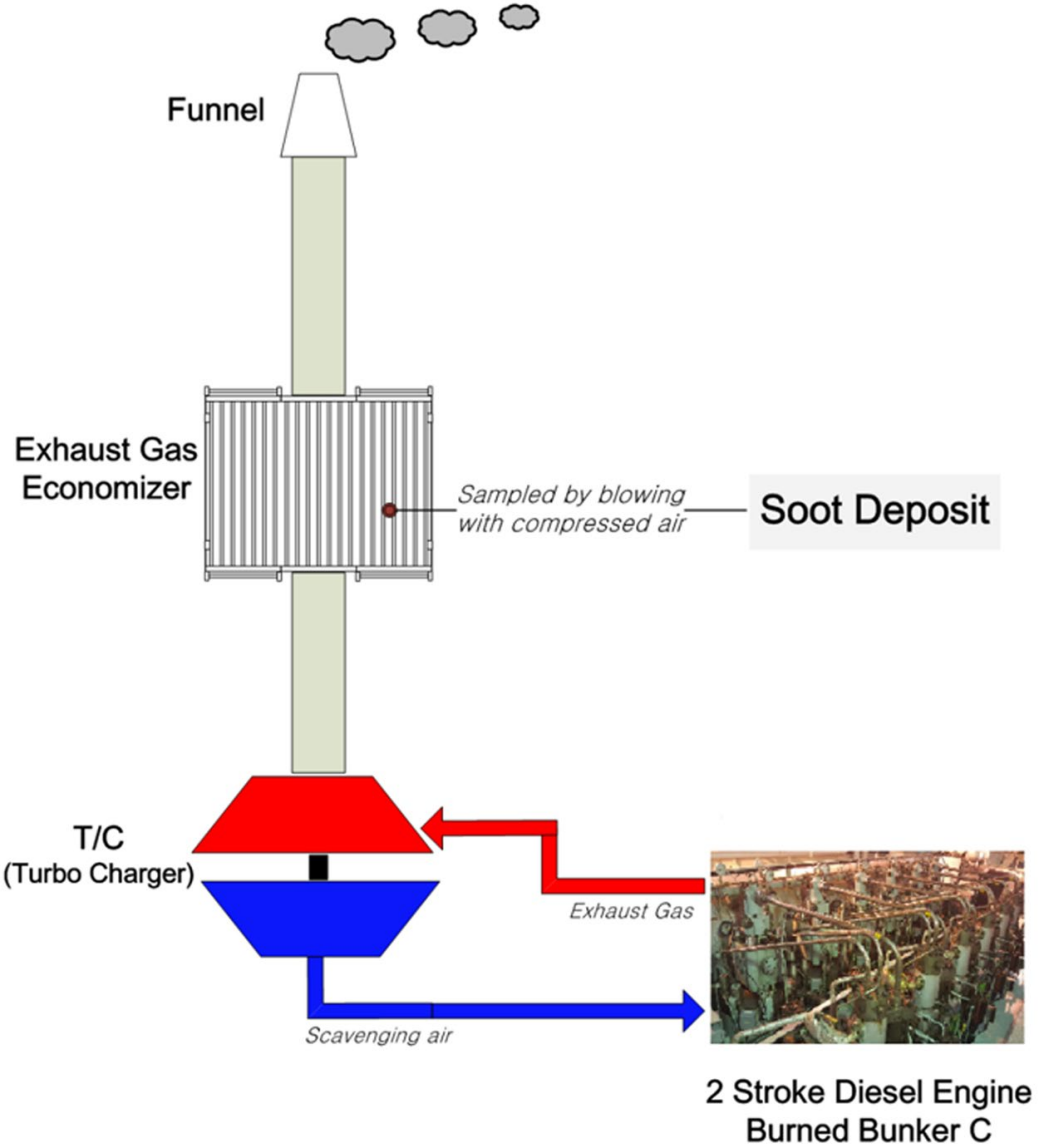
Schematic of soot sampling.

Soot was collected from container ships currently in operation. Detailed specifications of the ship and its engine are shown in Tables 1 and 2. Note that soot can be generated from various machines in the engine room of the ship; in this study, we collected soot from the economizer, where the largest amount of soot accumulates. The schematics of the economizer and the specifications of the bunker fuel oil that is the precursor of the soot are shown in Fig. 1 and Table 3, respectively.

**Table 1:** Technical description of the container ship (M/V SUNNY SPRUCE).

Items	Description
Vessel Name	M/V SUNNY SPRUCE
Gross Tonnage	3,981 MT
Length Overall	107.3 m
Breath	17.2 m
Maximum Speed	16.78 knots
Engine Model	MAN B&W 7L35MC
Output X RPM (MCR)	5,320 PS × 200 RPM
F.O. Consump. (at sea)	HFO: 15.3 MT (at NCR)
Kind of Fuel Oil	IFO 380 cSt

**Table 2:** Engine specifications.

Equipment	Items	Specification
2-stroke diesel engine	Manufacturer	MAN Diesel & Turbo
	Model	7L35MC
	MCR	5,320 PS x 200 rpm

**Table 3:** Fuel oil specifications.

Parameters	Unit	Results
Specific gravity @15/4 °C	—	0.9867
Viscosity Kin. @50 °C	mm^2^/s	321.3
Flash point	°C	74
Sulfur content	Weight %	2.89
Water sediment	Volume %	0.05

### Graphitization procedure

In the graphitization procedure, 10 g of soot was placed in an ultra-high temperature furnace (Thermvac Engineering, Korea) and heated to 2700 °C (to ~1800 °C at 10 °C/min, then to ~2400 °C at 5 °C/min, and finally to ~2700 °C at 3 °C/min). The soot was held at this temperature for 2 h under a flow of Ar gas (4 L/min). The furnace was allowed to cool naturally to ambient temperature, yielding the annealed soot.

### Carbon characterization

The morphology of the soot was investigated by transmission electron microscopy (TEM) (JEM-2100F; JEOL, Japan) at an acceleration voltage of 200 kV. XRD profiles (XRD, D8 Discover, BRUKER, German) were obtained using Cu K_α_ (λ = 1.540598 nm) as a target in the 2θ range of 10–90° with a step size of 0.02° and a scan speed of 2° min^−1^. Raman spectra were recorded using a Thermo Fisher Scientific Raman spectrometer (Thermo Fisher Scientific, USA) with a laser excitation wavelength of 532 nm. BET surface areas were calculated from N_2_ adsorption-desorption isotherms obtained using a Quantachrome sorption analyser (Autosorb-1, USA). Prior to BET measurement, all samples were subjected to heat treatment for 2 h at 200 °C under N_2_ to remove moisture. Thermogravimetric analysis (TGA) were performed using a TGA Q500 (TA Instrument, England) under atmosphere to determine the weight of the residue in the annealed soot. The elemental analysis of waste soot was carried using a Carbon Hydrogen Nitrogen Sulphur (CHNS) analyzer (Thermo Fisher Scientific, EA1112, USA) to determine the percentage composition of elements present in it.

### Electrochemical measurements

For the electrochemical evaluation of soot, an anode slurry was prepared by mixing soot (80 wt%), carbon black (10 wt%; Super P) as a conducting agent, and CMC/SBR (10 wt%) dissolved in distilled water as a binder. The slurry was coated onto Cu-foil substrates using a doctor blade coater and dried for 12 h at 50 °C under vacuum. CR2032-type coin cells were fabricated in a glovebox filled with Ar using Li coin chips as the counter and reference electrodes, Celgard 2400 as the separator, and 1 M LiPF_6_ in ethylene carbonate (EC)/diethyl carbonate (DEC) (1:1, v/v) containing 10 wt% fluoroethylene carbonate (FEC) as the electrolyte. All the coin cells were galvanostatically charged and discharged between 0.05 and 3 V (vs Li^+^/Li) at a current density of 1 C (372 mAg^−1^) using a Biologic BCS 805 Battery Test System at room temperature.

## Results and Discussion

**Figure 2:**
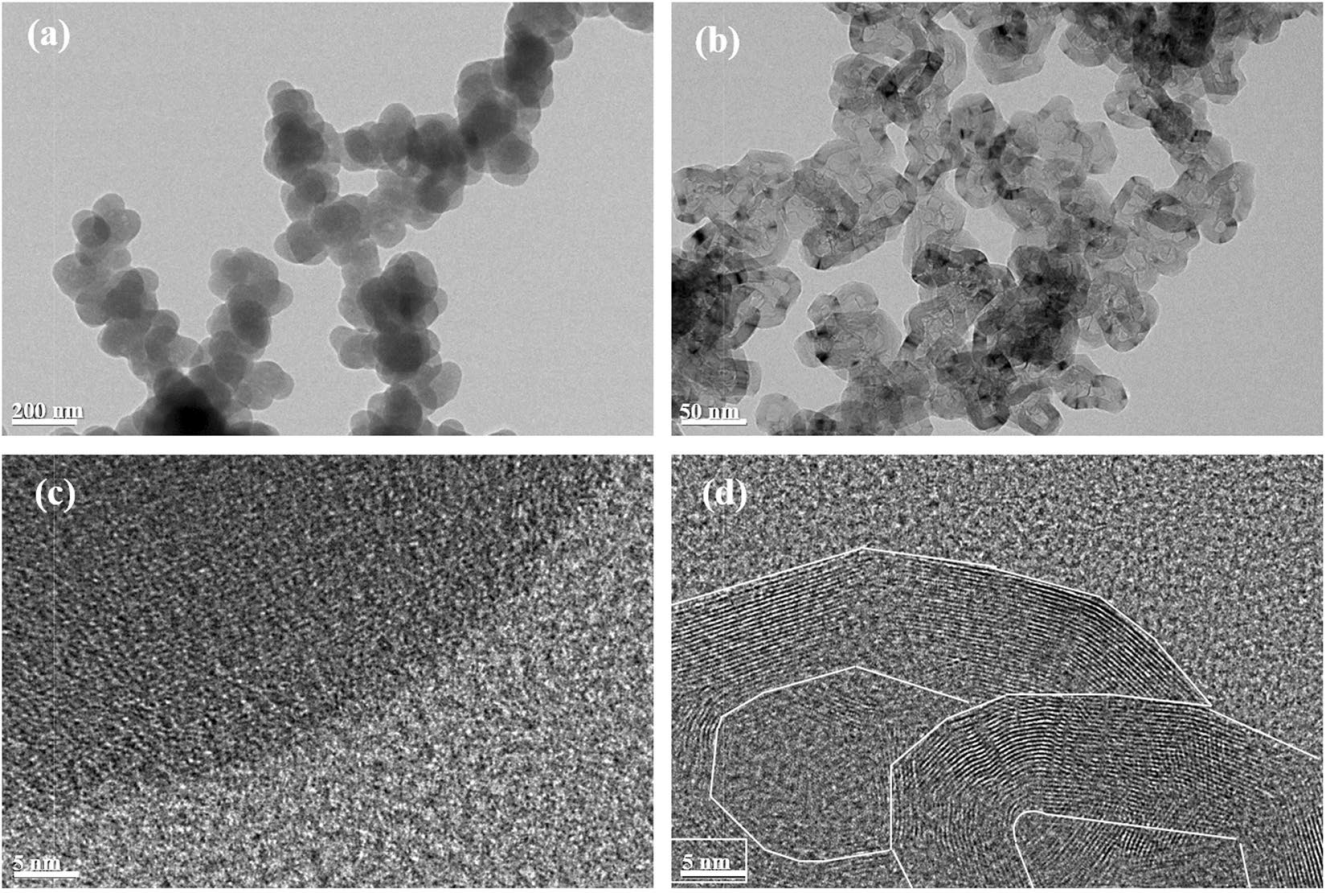
TEM images of soot: (**a**) as-obtained soot, (**b**) annealed soot. HR-TEM images of soot (**c**) as-obtained soot, (**d**) annealed soot.

The morphology of the soot before and after graphitization was observed by TEM. The images show that the shape of the soot is typical of carbon black. The primary particles of the agglomerated soot ranged from 70–100 nm in size with a relatively regular size distribution, and aggregated in different directions to form inter-connected structures with chain-like morphologies. The TEM images show a significant change after heat treatment, with the soot changing to an amorphous graphite-like structure (Fig. 2). Prior to heat treatment, the raw soot showed a typical disordered amorphous structure. On the other hand, after treatment at 2700 °C, the layered packets were found to be parallel to the concentric direction, and a stiff, flat, lamellar plane around an irregular or hollow core with a diameter of ~20 nm was apparent throughout the sample. This indicates that the layers grew significantly with increasing heat treatment temperature (HTT) and changed to an almost perfectly crystalline graphite structure. This type of carbon is known as carbon nano‐onions (CNOs); however, the diameters seen here are much larger than that generally observed for CNOs (20–30 nm). On the other hand, the TEM images also shows that the size of the carbon particle after the heat treatment is reduced. The waste soot contains a very large amount of hydrogen and sulfur before heat treatment (Table 4). These elements make the carbon structure very disordered structure. However, after the heat treatment at 2700 °C, these elements were not detected. For this reason, the structure of carbon changes from turbostratic structure to perfect graphite and thus d-spacing of graphite is also greatly reduced. Therefore, it is presumed that the degassing of hydrogen and sulphur, and the reduction of d-spacing will lead to a reduction in the size of carbon particles.

**Table 4:** CHNS elements analysis results of waste soot (wt%).

sample	Carbon	Hydrogen	Nitrogen	Sulfur
as-obtained soot	79.83	1.20	0.77	3.16
annealed soot	98.64	not detected	not detected	not detected

**Figure 3:**
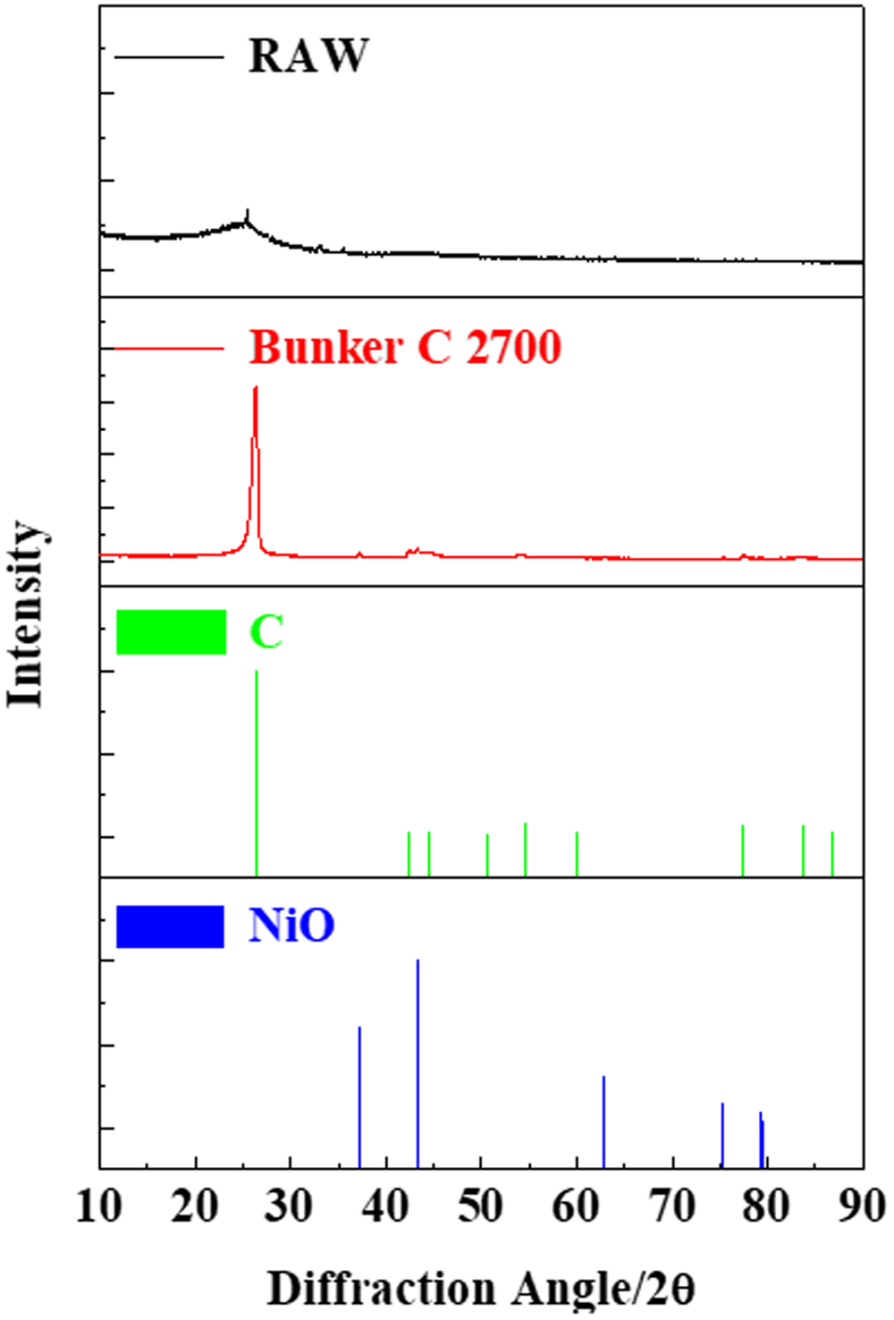
X-ray diffraction profiles of raw soot and annealed soot.

XRD profiles were measured in order to confirm the change in crystallinity. The obtained XRD profiles (Fig. 3) were corrected for the background baseline and instrumental broadening to ensure accurate microstructural characterization. The average interlayer spacing was calculated from the corrected position of the (002) peak using Bragg’s equation. The interlayer spacing changed from 0.350 to 0.338 nm after heat treatment, indicating that the turbostratic (fully disordered) soot structures converted into ordered structures. However, the interlayer spacing was still slightly larger than the theoretical value for crystalline graphite (0.3354 nm), indicating that the soot was not perfectly graphitized after heat treatment. The layer stacking height (L_c_) was calculated from the (002) peaks using Scherrer’s formula^27^. The L_c_ increased from 9.57 to 16.64 nm after heat treatment, with the 2θ value corresponding to the (002) peak shifting from 25.38° to 26.25° and becoming narrower. This shows that the height of the stacked graphite layers increased as the so-called aromatic sheets were built and ordered. Furthermore, the basal plane length (L_a_) was obtained from the (100) peaks using Scherrer’s formula^27^. The L_a_ increased from 19.71 to 35.1 nm after heat treatment, indicating noticeable ordering of the aromatic nanoclusters in the parallel direction. The observed increase in height (L_c_) and the large increase in lateral crystallite dimension (L_a_) indicate that the graphitized structure extended further in the direction of the plane than it was stacked in the direction perpendicular to the plane.

**Figure 4:**
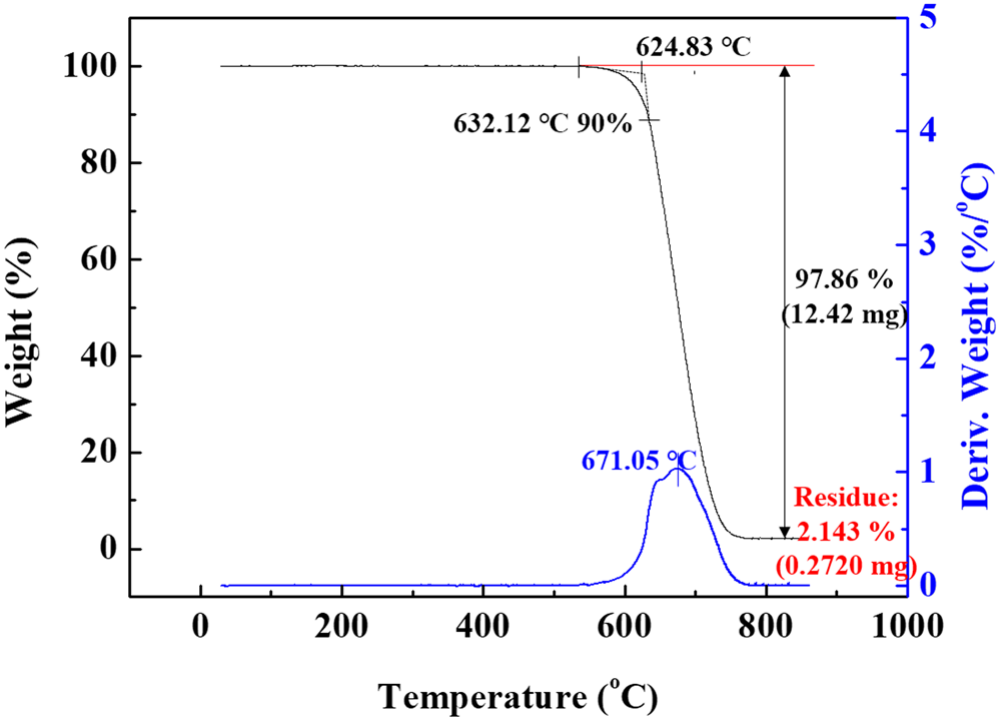
Thermogravimetric analysis graphs of annealed soot.

On the other hand, the XRD profile shows that the soot contained various impurities before the heat treatment; however, most of these impurities disappeared after the heat treatment and only the carbon peaks remained. However, some small peaks appeared after heat treatment, presumably corresponding to Ni oxide, which is used as a desulfurization catalyst in bunker fuel oil. The content of NiO was analysed by TGA, and it was found that annealed soot contained a very small amount of NiO (Fig. 4).

**Figure 5:**
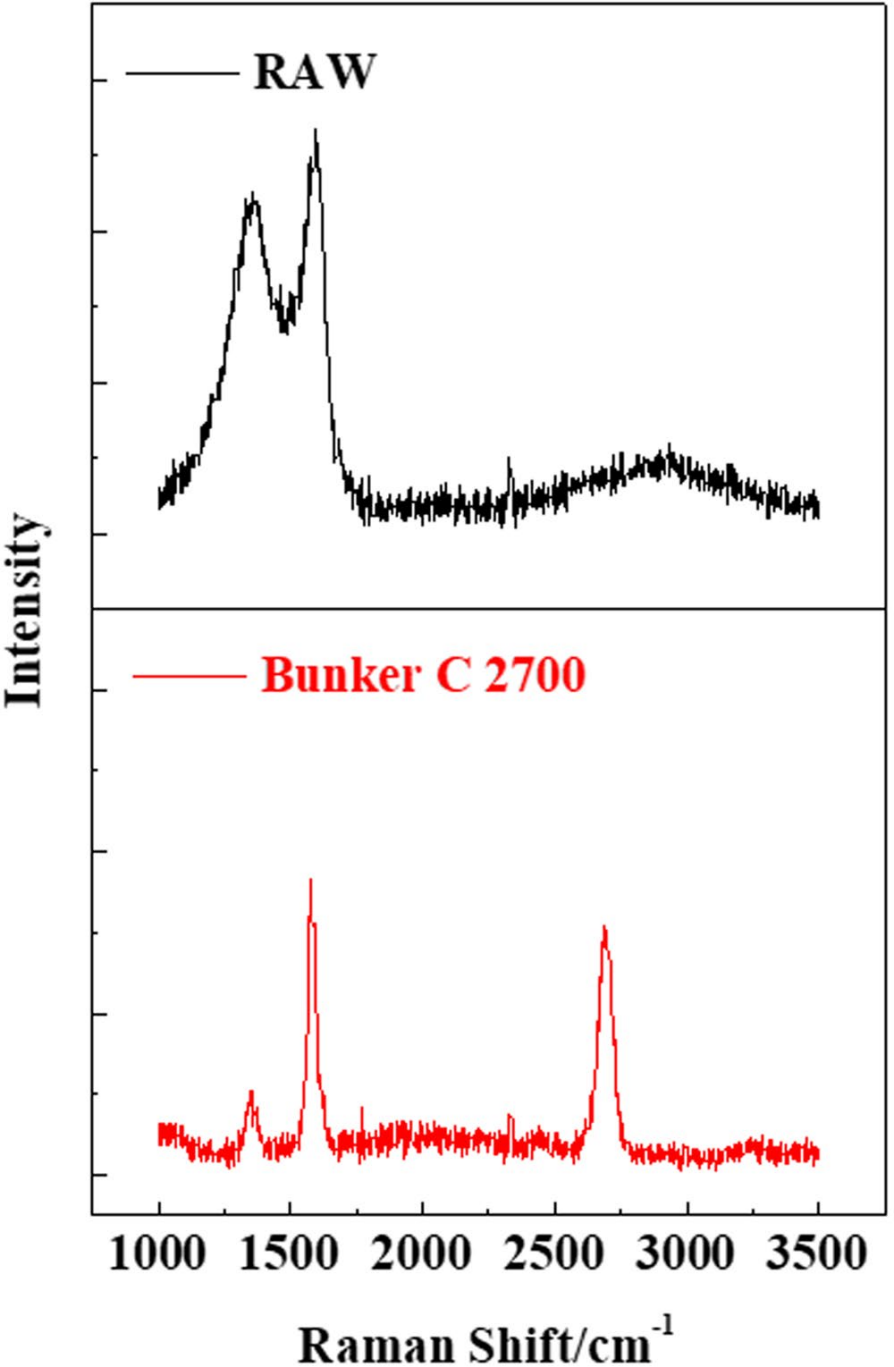
Raman spectra of raw soot and annealed soot.

Raman spectroscopy was performed to study the crystalline features of the annealed soot in detail (Fig. 5). The most dominant and characteristic Raman features in graphitic materials are the so-called D band (~1350 cm^−1^), G band (~1582 cm^−1^), and 2D band (~2700 cm^−1^). The D band originates from the presence of disorder in sp^2^-hybridized carbon systems associated with graphene edges, and is therefore known as the disordered or defect mode^28^. The G band arises from the stretching of the C-C bond in graphitic materials, and is common to all sp^2^ carbon-containing systems^28^. Thus, the ratio of the intensity of the G band to the D band (I_D_/I_G_) is widely used to evaluate the crystal purity and defect concentration in graphitic materials^29^. The I_D_/I_G_ ratio of the soot sharply decreased from 0.9 to 0.24 after heat treatment, and was inversely proportional to the in-plane dimension of the crystallites (L_a_). In addition, the G band shifted toward a lower frequency (from ~1588 to ~1580 cm^−1^, the theoretical value for graphite) after heat treatment. These results imply a high degree of graphitization, resulting in the graphitic order of the annealed soot, further supporting the conclusions drawn from the HRTEM and XRD results.

On the other hand, the 2D band frequency is strongly influenced by the number of layers in the graphite. Interactions between stacked graphene layers tend to shift this band to higher frequencies^30^. The 2D band of the annealed soot appeared at 2700 cm^−1^, indicating the presence of graphite. In addition, the shape of the 2D peak shows that high-quality graphite was formed because damaged graphene (or graphene oxide) yields very broad and low-intensity 2D peaks.

**Figure 6:**
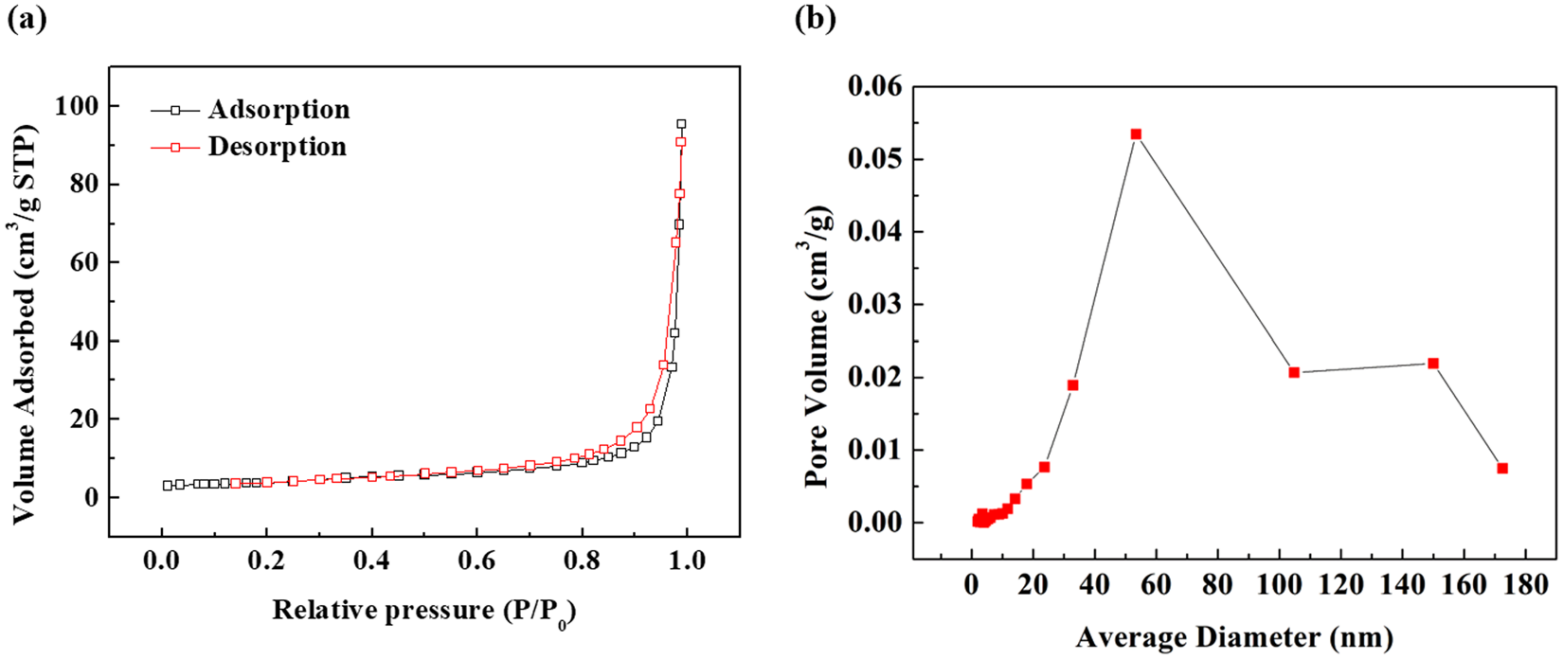
Nitrogen adsorption-desorption isotherms of annealed soot and pore size distribution.

The nitrogen adsorption/desorption isotherms for the annealed soot are displayed in Fig. 6a and the results summarized in Table 5. A linear BET range of 0.05–0.35 was used, and the BET surface area of the raw soot was calculated as 8.2 m^2^g^−1^. Generally, the graphite used in LIBs is micron-scaled, and thus, its BET surface area is very low (less than 2 m^2^g^−1^). However, soot takes the form of carbon black, which has nanoscale primary particles that lead to a high specific surface area. The BET surface area of the annealed soot was calculated as 13.3 m^2^g^−1^; this increase in surface area is potentially due to the removal of hydrogen from soot during the annealing process. High surface areas (i.e., smaller anode particles) are beneficial for quicker charging of LIBs because it allows high conduction rates. However, it can also cause low 1^st^ cycle efficiency due to consumption of Li by the initial formation of solid electrolyte interphase (SEI) layers on the carbon surface, although this drawback could be overcome by using a prelithiation process^31^.

**Table 5:** Results of BET measurements.

sample	BET (m^2^/g)	Pore Volume (m^2^/g)
as-obtained soot	8.2	0.10
annealed soot	13.3	0.15

Figure 6b Shows the pore size distribution (PSD) analyzed based on isotherm. The PSD shows that soot is a meso-macro hierarchical structure. This can be explained as follows. The primary soot particles are arranged into 100–300 nm agglomerates. The agglomerates are aggregated into a chained aggregate and a continuous pore network (>20 nm) is formed in the interstices. That is, as the soot primary particles agglomerate and aggregate into larger units of a few micrometers, they are creating an extensive porous network. A similar character of particle aggregation is observed for conductive carbon black such as Ketjen Black and Vulcan XC-72. This property shows that if the electric conductivity of soot is ensured by heat treatment., it can be fully utilized as a conductive material. This will be discussed in the electrochemical analysis part.

**Figure 7:**
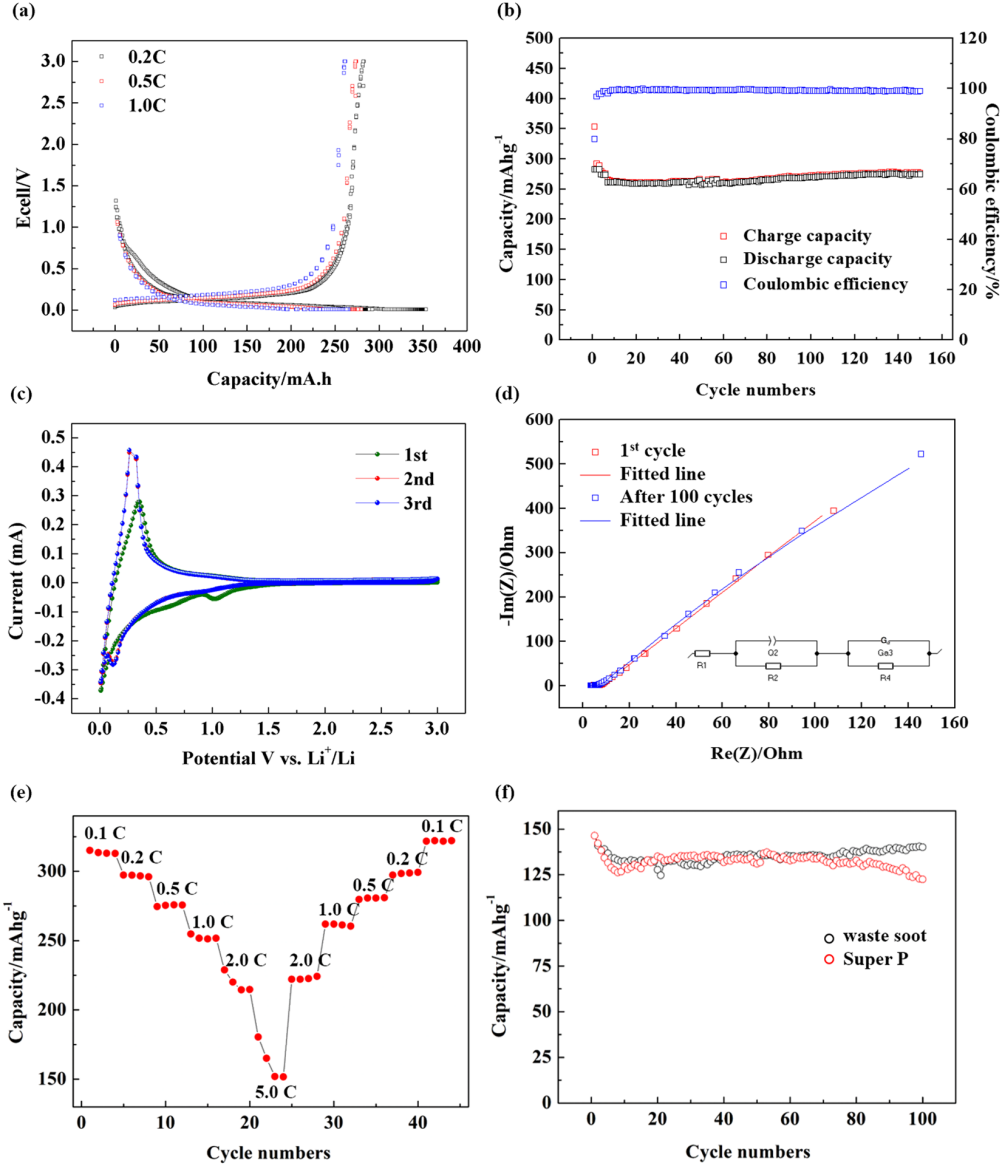
(**a**) Galvanostatic discharge/charge curves (**b**) cycling performance and columbic efficiency of the annealed soot cycled at a rate of 1 C (**c**) CV curves of the annealed soot electrode at a scanning rate of 0.2 mV s −1 in the voltage range of 0.005–3 V (vs. Li/Li^+^) (**d**) EIS of the annealed soot at different cycles (**e**) rate capability of annealed soot (**f**) comparison of cycling performance as conductive material with Super P.

Galvanostatic charge/discharge experiments were performed to evaluate the electrochemical performance of the annealed soot as an anode material for LIBs. Figure 7a shows the charge/discharge curves of the annealed soot over the first three cycles at rates of C/5, C/2, and C. The calculated reversible capacities were 282, 273, and 261 mAhg^−1^, respectively, and the material exhibited excellent output characteristics as the C rate was increased. In general, the amount of energy that can be extracted from a battery decreases with increasing discharge current due to an increase in the internal impedance of the battery. However, the capacity fade was very low when the soot was used, potentially because Li ions could be inserted and removed more easily with increasing specific surface area and via the graphitic edges exposed to the outside through the graphitization process. Meanwhile, an irreversible capacity of about 70.9 mAhg^−1^ was observed owing to the large specific surface area for the annealed soot during the first charge/discharge process. However, after the third cycle, the Coulombic efficiency was >95%; this initial irreversible capacity could be significantly decreased by performing prelithiation during the actual production process. Furthermore, the soot exhibited a good cycle life and reversibility after long-term cycling over 150 cycles at a rate of 1 C (Fig. 7b). After 150 cycles, the soot anode still showed a specific reversible capacity of 260 mAhg^−1^. In addition, the columbic efficiency of the waste soot electrode after the first 3 cycles kept around ∼99% until 150 cycles, suggesting the highly reversible Li+ insertion/extraction kinetics^32^.

Cyclic voltammetry was performed to examine the reduction and oxidation peaks in the voltage range of 0.01–3.0 V (vs. Li/Li+) at a scan rate of 0.2 mV/s using the same workstation. Figure 7c exhibits the first three consecutive cyclic voltammetry (CV) curves of the annealed soot anode consisting of three distinct reduction peaks. The first peak located at 1.1 V could be assigned to the irreversible reduction of fluoroethylene carbonates (FEC). The second broad cathodic peak from 0.25–1.1 V corresponds to ethylene carbonate and dimethyl carbonate (EC/DEC) decomposition and the formation of a solid electrolyte interphase (SEI) layer. Finally, a sharp peak indicating the insertion of Li ion is observed at 0.25 V or less. After the first cycle, the cathodic reduction peaks disappear, and the CV curves nearly overlap without any obvious changes in the magnitude of the peak current or potential. This indicates the good reversibility of the Li insertion and extraction reactions and the cycling stability of the annealed soot anode^33^. In the anodic reaction, it shows that most lithium is delithiated at voltages below 1.5 V. These results show the potential of soot as a promising candidate for producing low cost anode materials for use in LIBs.

The impedance measurements were carried out to study the resistance of the SEI film and charge transfer resistance at different cycling. In Fig. 7d, the experimental data (indicated by dots) and simulated data (indicated by line) for annealed soot is shown at different cycles. The impedance spectra observed consists of a semicircle at the high frequency end. Information regarding solution, surface film resistances can be obtained from the semicircle at the high frequency end^34^. The depressed nature of the semicircle can be attributed to the merging of the two different semicircles. One is due to the surface film and the other, is from the charge-transfer process. The diameter of the annealed soot semicircle is clearly small, which means that the resistance is very small. It maintains the same value after the 50th cycle, suggesting that the SEI film is stable and charge transfer resistance does not increase. This is consistent with the CV results, in which no further decomposition of the electrolyte occurred after the SEI was formed stably.

The rate capability of the annealed soot is shown in Fig. 7e. The initial high capacity of 315 mAhg^−1^ was observed at a current density of 0.1 C after four discharge/charge cycles. The capacity of the annealed soot was measured to be 315, 297, 275, 251, 218 and 150 mAhg^−1^ when the current rate was consecutively set at the levels of 0.1 C, 0.2 C, 0.5 C, 1 C, 2 C and 5 C. As the current density was reduced to a low current, the capacity also recovered completely. A capacity of 320 mAhg^−1^ was detected in the 40th cycle when the current rate was returned to the value of 0.1 C. This result indicates that the structure of the annealed soot is stable at various current densities.

Meanwhile, in order to find other commercial applications of waste soot, we used waste soot as a conductive material after heat treatment at 2000 °C for 2 h. Figure 7f shows a cycling performance by using artificial graphite as the anode active material and using the conventional commercial conductive material (Super P) and waste soot as the conductive material, respectively. The artificial graphite and Super P were all purchased from MTI Corporation. The slurry recipe and coating process were carried out completely with the procedures introduced in the experimental method. The performance of the two cells was not significantly different, indicating that the waste soot can be fully utilized as a conductive material by heat treatment at 2000 °C. These results show the potential of soot as a promising candidate for producing low cost anode materials and conductive material for use in LIBs.

## Conclusion

This study represents the first attempt to recycle waste soot from ships into an active material for use in LIBs, which is a unique idea of utilizing waste for producing renewable energy. Although soot is generated from various machines on the ship, the soot used in this study was collected from the economizer as it generated the maximum quantity; this rendered it most suitable for potential mass production. The collected soot was graphitized through heat treatment at 2700 °C to enable its use as an anode active material. The morphology and structure of the obtained soot were investigated by HR-TEM, which revealed that the graphitized soot formed CNOs; however, these were larger than normal nano-onions. From the XRD, Raman spectroscopy, and BET surface area results, it was confirmed that the graphitized soot was converted into highly crystalline graphite, and the specific surface area of the graphitized soot was slightly higher than that generally used in active materials.

The annealed soot with a unique graphitic multilayer structure had an electrochemical performance that rendered it suitable as a candidate for anode materials. In addition, it has a high reversible capacity and good cycling performance, which are critical for rechargeable LIBs. It will be necessary to carry out the same analysis and research for other types of soot emitted from ships in the future, and to conduct research to find various uses for waste soot.
